# FLi_4_X_4_^−^ (X = Cl, Br, I): Superhalogen Anions with Planar Tetracoordinate Fluorine

**DOI:** 10.3390/molecules29235810

**Published:** 2024-12-09

**Authors:** Yong-Xia Li, Li-Xia Bai, Jin-Chang Guo

**Affiliations:** 1 Department of Chemistry, Xinzhou Normal University, Xinzhou 034000, China; 2 Institute of Molecular Science, Shanxi University, Taiyuan 030006, China; bailixia2021@163.com

**Keywords:** planar tetracoordinate fluorine, superhalogen anions, global minimum, multicenter ionic bonding, stability

## Abstract

The concept of superhalogen was proposed for more than 40 years, and it has never been associated with planar tetracoordinate fluorine (ptF) species. In this work, using Li as the ligands and Cl, Br, I as the auxiliary atoms, we have designed the star-like *D*_4*h*_ FLi_4_X_4_^−^ (X = Cl, Br, I) clusters, which contain the ptF at the centers. They are all global minima (GMs) based on unbiased searches on the potential energy surfaces. Born–Oppenheimer molecular dynamics (BOMD) simulations suggest that these ptF structures are robust against dissociation at room temperature. Chemical bonding analyses indicate that there are four lone pairs (LPs) for ptF, three LPs for each X atom, and four 3c-2e Li–X–Li σ bonds. The stabilities of these ptF clusters are dominated by multicenter ionic bonding, rather than the σ aromaticity. Interestingly, these ptF species have large first vertical detachment energies (7.37, 6.94, and 6.30 eV). According to the definition of superhalogen, they can be viewed as superhalogen anions. The current work builds an important link between superhalogen and ptF chemistry.

## 1. Introduction

In 1874, van’t Hoff and Le Bell independently proposed the concept of tetrahedral carbon (thC), which has dominated organic chemistry for 150 years [1,2]. The non-classical bonding of carbon has attracted much attention from both theoretical and experimental researchers in recent decades. Planar tetracoordinate carbon (ptC) was first mentioned in a hypothetical transition state structure by Monkhorst in 1968 [3]. How to stabilize the ptC species? Two years later, Hoffmann and coworkers proposed the “electronic” and “mechanical” strategies, which have been a general guide to designing the new ptC compounds [4]. Using the electronic strategy, the first viable ptC cluster 1,1-dilithiocyclopropane (C_3_H_4_Li_2_) was theoretically predicted by Schleyer et al. in 1976 [5]. Note that it is only a local minimum on the potential energy surfaces. Since then, the project of stabilizing planar carbon, including the planar tetra-, penta-, and hexa-coordinate carbons (ptC, ppC, and p6C), has intrigued theoretical and experimental chemists for nearly 50 years [6,7,8,9,10,11]. Among the reported ptC species, several aluminum-based ptC clusters (CAl_4_^−^, NaCAl_4_^−^, CAl_3_Si^−^, CAl_3_Ge^−^, CAl_4_H^−^, CAl_11_^−^, and C_5_Al_5_^−^) stood out, which were detected in the gas-phase photoelectron spectroscopy (PES) experiments [12,13,14,15,16,17,18]. The ppC and p6C global minima (GMs) represented by CAl_5_^+^ and CE_3_M_3_^+^ (E = S, Se, Te and M = Li, Na, K, Rb, Cs) have been theoretically predicted and await further experimental characterization [19,20].

The concept of planar hypercoordinate carbon (phC) can be further extended to other inorganic, main-group, and transition-metal atoms in the periodic table. A large family of planar hypercoordinate atoms has been formed and is still growing. As early as 1991, Schleyer and Boldyrev proposed a general strategy for achieving planar tetracoordinate geometries for carbon and other second-row periodic elements [21]. They predicted ptC cis-CA1_2_Si_2_, trans-CA1_2_Si_2_, ptO OAl_4_, ptN NAl_4_^−^, NAl_3_Si, and ptB BAlSi_3_ clusters. In 2000, the ptSi and ptGe were observed in the pentatomic SiAl_4_^−^ and GeAl_4_^−^ clusters by Boldyrev and Wang [22]. Recently, Zheng et al. identified the ptSi Si_3_Cu_3_^−^ GM structure by using the PES technique [23]. Seven clusters (LiNa_5_, Li_5_Mg^+^, Na_5_Mg^+^, K_5_Ca^+^, CaRb_5_^+^, Rb_5_Sr^+^, and SrCs_5_^+^) with a planar pentacoordinate s-block metal as the GMs were reported by Cui and coworkers [24]. As the smallest atom in the periodic table, H also joins the planar hypercoordination family. Recently, the ptH HIn_4_^+^, HLi_4_H_3_^−^, HK_4_H_4_^−^, and ppH HLi_5_H_5_^−^ clusters were reported, which initiated the phH chemistry [25,26,27,28].

As the most electronegative element in the periodic table, the coordination number of fluorine atoms is usually no more than 3. In 2021, Merino et al. predicted the first series of ptF clusters including FIn_4_^+^, FTl_4_^+^, FGaIn_3_^+^, FIn_2_Tl_2_^+^, FIn_3_Tl^+^, and FInTl_3_^+^ [29]. Recently, Guha et al. predicted the doublet ptF FM_4_H_3_^−^ (M = Li, Na, K) GM clusters [30]. As a new research focus, ptF-chemistry mainly focuses on the stability and bonding of compounds. Very recently, the square-like ptF FK_4_H_4_^−^ GM was reported by our group [31]. Interestingly, its first vertical detachment energy (VDE, 3.57 eV) is close to the electron affinity (EA) of Cl (3.61 eV) and can be seen as a pseudo-halogen anion. It built an important link between ptF structure and pseudo-halogen character.

The EA can describe the ability of an atom to gain an electron. In the periodic table, the element with the highest electron affinity is chlorine. The EA of clusters can overcome this limit due to the collective effects. The concept of superhalogen was proposed by Gutsev and Boldyrev in 1981, which refers to a class of species with greater VDE than the EA of halogen or their anions [32]. A general formula MX_k+1_ (M is a central electropositive atom with valence k and X is electronegative ligands) was also proposed by them to design the superhalogens. Since then, a large number of superhalogens and their anions have been reported theoretically or experimentally [33]. Most of them are collected in a recent review. Notably, the first series of superhalogen anions MX_2_^−^ (M = Li, Na; X = Cl, Br, I) were observed in the PES experiments by Boldyrev and Wang in 1999 [34]. In 2004, they identified a series of anionic sodium chloride clusters, Na_x_Cl_x+1_^−^ (X = 1–4), using an ab initio genetic algorithm and photoelectron spectroscopy [35]. The VDEs for Na_x_Cl_x+1_^−^ were measured to be 5.6, 6.46, 6.3, and 7.0 eV, respectively, which can be viewed as the superhalogen anions. Among the reported superhalogens or their anions, there are no ptF species.

In this work, using Li as the ligands and Cl, Br, I as outer auxiliary bridges, we have designed the ternary ptF FLi_4_X_4_^−^ (X = Cl, Br, I) species, which are all GM structures based on extensive searches on the potential energy surfaces. Encouragingly, the ptF FLi_4_X_4_^−^ (X = Cl, Br, I) clusters have the large first VDE values (7.37, 6.94, 6.30 eV) at the single-point CCSD(T) level. According to the superhalogen concept, they can be regarded as superhalogen anions. The unique ptF structures, coupled with the superhalogen anion properties, make the FLi_4_X_4_^−^ (X = Cl, Br, I) clusters have potential applications. These novel clusters are expected to be synthesized and characterized experimentally, which will further expand the research fields of ptF chemistry and superhalogens. This finding highlights the factor of auxiliary atoms in the design of ptF species.

## 2. Results and Discussion

### 2.1. Structures and Stability

As the literature indicated, the ptF FLi_4_H_3_^−^ (*C*_2_*_v_*) is a true GM structure [30]. Intuitively, it can be changed from an open shell to a more symmetric closed shell structure by adding an H auxiliary atom. At the PBE0-D3(BJ)/def2-TZVPP level, *D*_4*h*_ FLi_4_H_4_^−^ is only a transition state structure, with one imaginary frequency (104i) [36,37,38]. The imaginary frequency corresponds to the a_2u_ vibrational mode, in which the central atom F moves up and down along the fourfold axis. Distortion of the *D*_4*h*_ structure leads to the *C*_4*v*_ minimum structure, in which the F atom lies about 0.54 Å above the Li_4_ plane. That is to say, the hollow Li_4_H_4_ ring is too small to hold the F atom in terms of geometry. How to flatten it? It is an effective method to increase the geometry of the ligand ring by replacing the peripheral auxiliary H atoms. Considering some similarities in bonding between H and halogen atoms, we designed the ptF FLi_4_X_4_^−^ (X = Cl, Br, I) (**1**–**3**) clusters by replacing the periphery hydrogen bridges in FLi_4_H_4_^−^ with halogen atoms. Note that ptF FLi_4_F_4_^−^ is only a transition state structure at the PBE0-D3(BJ)/def2-TZVPP level, due to the small Li_4_F_4_ ligand ring. Geometrically, the Li_4_X_4_ (X = Cl, Br, I) rings are suitable to hold one central ptF atom. Are these novel ptF clusters GM structures on the potential energy surfaces of the systems? The unbiased GM searches give a positive answer.

**Figure 1:**
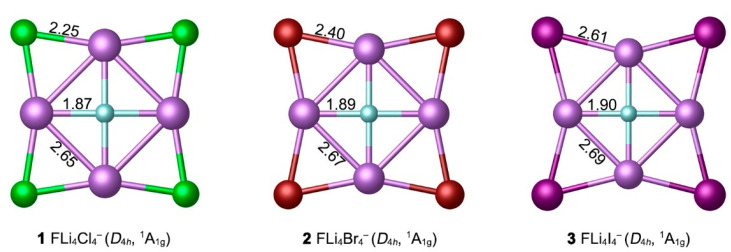
Optimized global minimum (GM) structures of the FLi_4_X_4_^−^ (X = Cl, Br, I) clusters at the PBE0-D3(BJ)/def2-TZVPP level. The bond distances are shown in Å.

**Figure 2:**
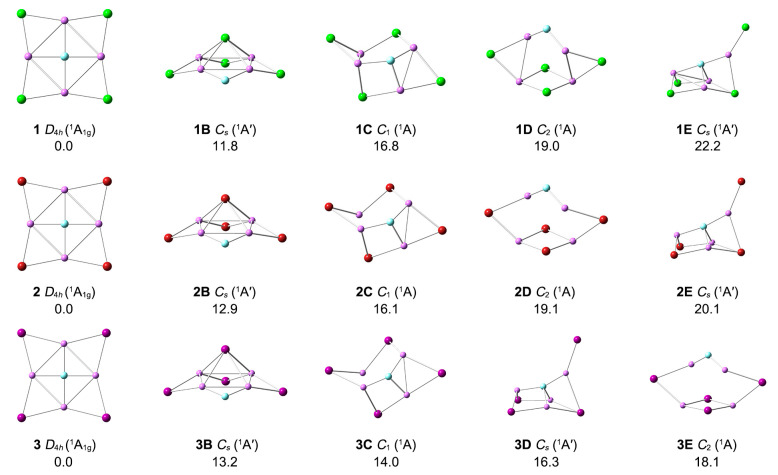
Optimized global-minimum structures **1**, **2, 3,** and their four low-lying isomers (***n*B**–***n*E**) at the PBE0-D3(BJ)/def2-TZVPP level. Relative energies are listed in kcal mol^−1^ at the single-point CCSD(T)/def2-TZVPP//PBE0-D3(BJ)/def2-TZVPP level, with zero-point energy (ZPE) corrections at PBE0-D3(BJ).

The optimized GM structures of FLi_4_X_4_^−^ (X = Cl, Br, I) are presented in Figure 1, along with the bond distances (in Å) at the PBE0-D3(BJ)/def2-TZVPP level. They possess star-like structures with high *D*_4*h*_ symmetry, composed of one FLi_4_ core and four outer X auxiliary bridges. As shown in Table S1, these ptF FLi_4_X_4_^−^ (X = Cl, Br, I) are true minima structures based on eight different theoretical levels. Their four alternative low-lying structures are shown in Figure 2. Their relative energies were determined at the single-point CCSD(T)/def2-TZVPP//PBE0-D3(BJ)/def2-TZVPP level, plus zero-point energy (ZPE) corrections at PBE0-D3(BJ)/def2-TZVPP [39]. The diagnosis of the T_1_ coupled-cluster operator was performed to verify whether the converged CCSD wave functions are based on a single-reference method. All the T_1_ values of the reported structures in this work are lower than the suggested threshold of 0.02. Structures **1**–**3** are reasonably well-defined GMs on the corresponding potential energy surfaces, being 11.8–13.2 kcal mol^−1^ more stable than the most competitive isomers at the single-point CCSD(T)/def2-TZVPP//PBE0-D3(BJ)/def2-TZVPP level. The isomers **1B**, **2B**, and **3B** can be obtained by swapping the central F atom with one peripheral X (Cl, Br, I) bridge atom. The fluorine atom, which is the most electronegative, tends to bond to all alkali metal Li atoms. Thus, the ptF atom is more beneficial than the μ^2^-F bridge to the energy of the system. Most X atoms are bonded to ligand atoms by bridging-μ^2^ way in **1**–**3E**. In these low-lying isomers, no terminal μ^1^-F atom was found. Due to the presence of the four alkali metals, halogen atoms are always bonded to alkali metals, and there is no bond between them. The <LiXLi bond angles in **1**–**3** are in the range of 61.8°–77.2°.

According to Kim et al., using high-level ab initio methods with large basis sets is needed for ptF species [40]. We also reoptimized the **1**–**3E** structures at the PBE0-D3(BJ)/aug-cc-pVQZ (aug-cc-pVQZ(PP) for I). As shown in Figure S1, **1B** is higher 10.6 kcal mol^−1^ than **1** at the CCSD(T)/aug-cc-pVQZ//PBE0-D3(BJ)/aug-cc-pVQZ level, which is close to that of CCSD(T)/def2-TZVPP//PBE0-D3(BJ)/def2-TZVPP. Taking **1** as an example, we also calculate the relative energies of structures with the central F atom at 0.1, 0.2, 0.3, and 0.4 Å above the plane, at the single point CCSD(T)/aug-cc-pVQZ level. As shown in Table S2, **1** has the lowest energy. Thus, the CCSD(T)/def2-TZVPP//PBE0-D3(BJ)/def2-TZVPP results are reliable.

In terms of bond distances, the F–Li (1.87, 1.89, and 1.90 Å) and Li–Li (2.65, 2.67, and 2.69 Å) distances in **1**–**3** are substantially similar, as the auxiliary halogen atom changes. The outer Li–X distances are 2.25, 2.40, and 2.61 Å, respectively, which are positively associated with the radius of the X atoms. The F–Li, Li–Li, and Li–X distances in **1**–**3** clusters are slightly shorter than the recommended lengths of covalent single bonds (F–Li 1.97, Li–Li 2.66, Li–Cl 2.32, Li–Br 2.47, and Li–I 2.66 Å) [41]. In such highly ionized clusters, single bond length data cannot truly reflect the corresponding bond strength.

**Figure 3:**
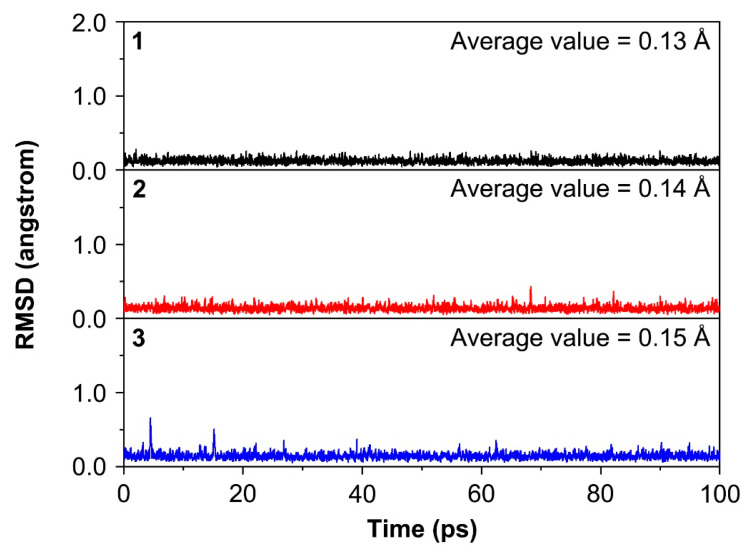
Calculated root-mean-square deviations (RMSDs) of GM clusters **1**–**3** during the Born–Oppenheimer molecular dynamics (BOMD) simulations at 298 K.

From the point of experimental characterization, to design ideal ptF clusters, it is necessary not only to have good thermodynamic stability but also to have excellent kinetic stability. The dynamic stabilities of **1**–**3** clusters were further evaluated by the Born–Oppenheimer molecular dynamic (BOMD) simulations at the PBE0/def2-SVP level, for 100 ps at 298 K [42]. As depicted in Figure 3, the root-mean-square deviation (RMSD) values’ curves in these simulations have no obvious leaps, suggesting these ptF structures are robust against the isomerization or dissociation. The average RMSD values are in the range of 0.13–0.15 Å, suggesting that the ptF **1**–**3** structures can be maintained at room temperature. As shown in Figure S2, the average absolute F1–Li2–Li3–Li4 dihedral angles are in the range of 8.34–8.77*°*, supporting the above conclusion.

**Figure 4:**
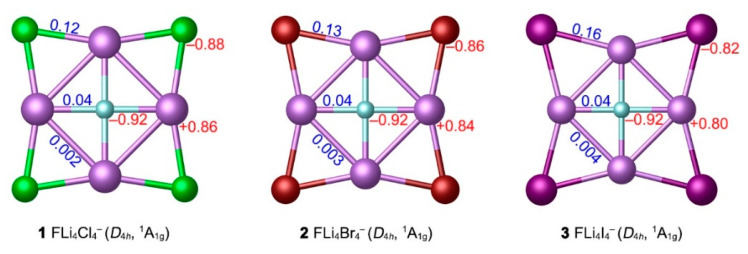
Wiberg bond indices (WBIs, in blue color) and atomic natural population analysis (NPA) charges (q, |e|, in red color) for **1**, **2, 3**.

The Wiberg bond indices (WBI) and natural population analysis (NPA) charges are listed in Figure 4, based on natural bond orbital (NBO) analysis [43]. The WBI_F–Li_ is only 0.04 in the ptF **1**–**3** clusters, indicating that there is little covalent bonding between them. The low WBI_Li–Li_ (0.002–0.004) values reveal that there is no covalent bonding between two adjacent lithium atoms. The WBI_Li–X_ (0.12–0.16) suggests that there is a certain degree of covalent bonding between the Li and periphery X atom. As the electronegativity of the X atom decreases, the covalence of the X–Li bond increases. The charge distributions of the atoms in these ptF clusters depend on their electronegativity differences. The electronegativities of halogen atoms are 3.98 (F), 3.16 (Cl), 2.95 (Br), and 2.66 (I), while that of alkali metal Li atom is only 0.95. The large electronegativity differences between different atoms make the ionization characteristics of the FLi_4_X_4_^−^ (X = Cl, Br, I) systems obvious. There are obvious charge transfers in these ptF systems. The F and Cl atoms in **1** carry negative charges of −0.92 and −0.88|e|, respectively, while Li possesses a significant positive charge of +0.86 |e|. The fluorine atoms in these ptF clusters become completely F^−^ anions due to the contribution of the Li ligand atoms. From an electrostatic point of view, such a negative–positive–negative charge distribution from the central atom to the ligand atom and the auxiliary atom is ideal for maintaining the stability of the system. The F and Cl atoms have the electron configurations [He]2s^1.95^2p_x_^1.99^2p_y_^1.99^2p_z_^2.00^ and [Ne]3s^1.97^3p_x_^1.95^3p_y_^1.95^3p_z_^1.99^, respectively, which satisfy the octet rule. The electrostatic interactions between the three regions help stabilize star-like ptF clusters. As depicted in Figure 4, the charge distributions of **2** and **3** are basically similar to the situation of **1**. Thus, the stabilities of the ptF **1**–**3** clusters are dominated by ionic bonding.

**Figure 5:**
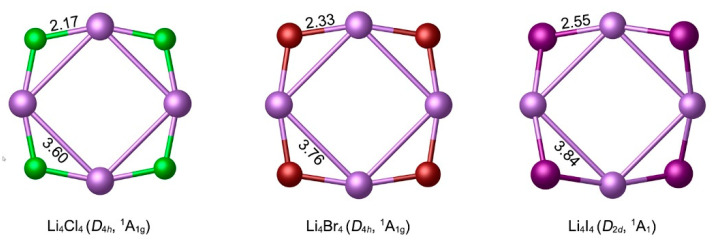
Optimized local minimum structures of the Li_4_X_4_ (X = Cl, Br, I) clusters at the PBE0-D3(BJ)/def2-TZVPP level. The bond distances are shown in Å.

The NPA charge data indicate that the FLi _4×4_^−^ (X = Cl, Br, I) clusters can be described as the ionic [F]^−^[Li_4_X_4_] or [F]^−^[Li_4_]^4+^[X_4_]^4−^ compounds. The hollow star-like Li_4_X_4_ (X = Cl, Br, I) rings are all true minima. It should be noted that Li_4_Cl_4_ and Li_4_Br_4_ possess the perfect *D*_4*h*_ symmetry, while Li_4_Br_4_ is slightly distorted to *D*_2*d*_ symmetry. The optimized structures of Li_4_X_4_ (X = Cl, Br, I) at the PBE0-D3(BJ)/def2-TZVPP level were depicted in Figure 5, with the bond distances (in Å). In the hollow Li_4_X_4_ (X = Cl, Br, I) structures, the Li–Li bond distances are in the range of 3.60–3.84 Å, which are obviously longer than those in the ptF **1**–**3** clusters. However, the Li–X bond distances (2.17–2.55 Å) in Li_4_X_4_ (X = Cl, Br, I) were slightly shorter than corresponding to those of **1**–**3** (2.25–2.61 Å). Thus, the addition of the ptF center weakens the bonding between the Li ligand and auxiliary X atoms and enhances the interaction between F and Li.

Concerning the thermodynamic stability of FLi_4_X_4_^−^ (X = Cl, Br, I), the energy changes of the following processes F^−^ + Li_4_X_4_ = FLi_4_X_4_^−^ were calculated. With zero-point corrections included, we find that the reactions are highly exothermic (∆E = −127.6, −135.4, and −142.5 kcal mol^−1^ for X = Cl, Br, and I, respectively), indicating that the desired ptF species are easy to form in thermodynamics. The central F^−^ anion and periphery Li_4_X_4_ rings can be viewed as the “core” and “shell”, respectively, which together form the unique planar core–shell structures of FLi_4_X_4_^−^ (X = Cl, Br, I). In addition, the calculated HOMO–LUMO energy gaps for the **1**–**3** anions are 7.49, 7.05, and 6.46 eV, further indicating the electronic stability of these ptF anions.

### 2.2. Bonding Characteristics

**Figure 6:**
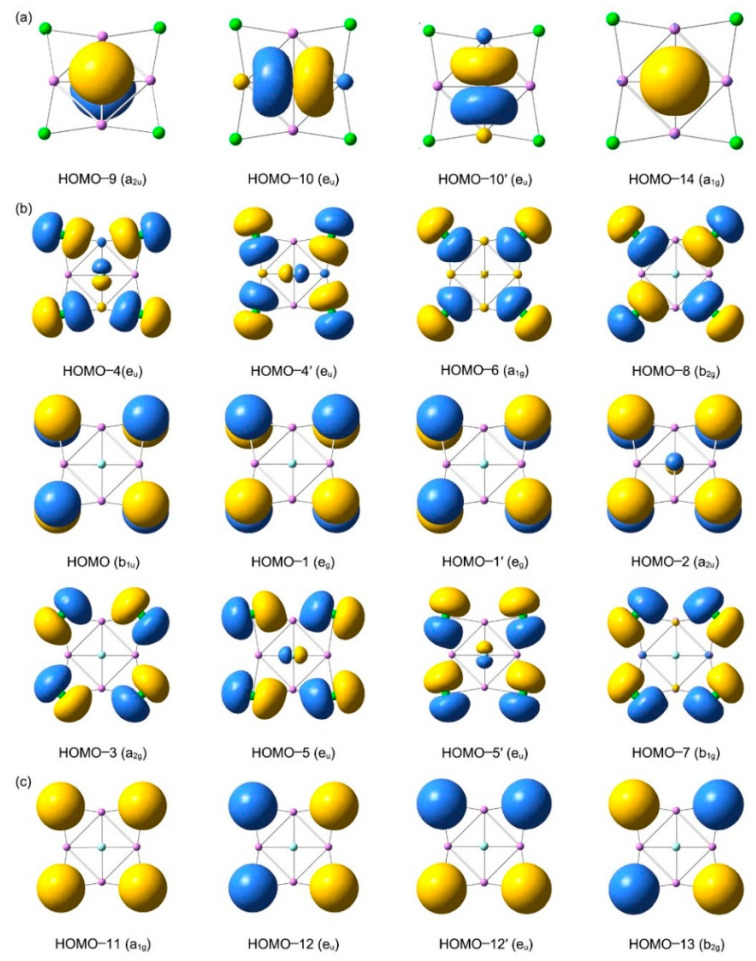
Analysis of canonical molecular orbitals (CMOs) of *D*_4*h*_ FLi_4_Cl_4_^−^ (**1**) cluster: (**a**) 4 CMOs for LPs of F, (**b**) 12 CMOs for LPs of four Cl atoms, and (**c**) 4 σ CMOs for delocalized Li–Cl–Li 3c-2e bonds.

Why are these ptF clusters so thermodynamically and dynamically stable? To understand the unique planar core–shell structures of GM **1**–**3** clusters, it is essential to carry out chemical bonding analyses. The chemical bonding in the ptF species may be understood via canonical molecular orbitals (CMOs) analysis, aided with orbital composition calculations. Let us analyze **1** as a representative case, because FLi_4_Cl_4_ and FLi_4_Br_4_, FLi_4_I_4_ are similar in geometries and bonding. Cluster **1** has 40 valence electrons (ve) (including one extra charge), which occupy 20 CMOs as depicted in Figure 6. The detailed CMO composition data of **1** are collected in Table S2. These CMOs can be sorted into three subsets, according to the types and components. Subset (a) involves 4 CMOs (HOMO-9, degenerate HOMO-10, and HOMO-14), primarily derived from F 2s, 2p_x_, 2p_y_, 2p_z_ atomic orbitals (AOs) in the molecular center. They can be classified as four lone pairs (LPs) of the ptF atom. Subset (b) includes 12 CMOs, which have substantial contributions from the outer four Cl atoms (3p_x_, 3p_y_, 3p_z_) and can be viewed as the LPs of four Cl atoms. Subset (c) has four CMOs (HOMO-11, degenerate HOMO-12, and HOMO-13), which are mainly derived from the s-type atom orbitals (Aos) of Li and Cl atoms. These CMOs are readily recombined as three-center two-electron (3c-2e) Li–Cl–Li σ bonds, which contribute to the rigidity of the outer Li_4_Cl_4_ ring. It should be noted that these orbitals have substantial contributions from the 3s orbitals of Cl atoms, from 93.3% to 96.7%. Four lithium atoms contribute only a little, because Li–X bonds are ionic.

**Figure 7:**
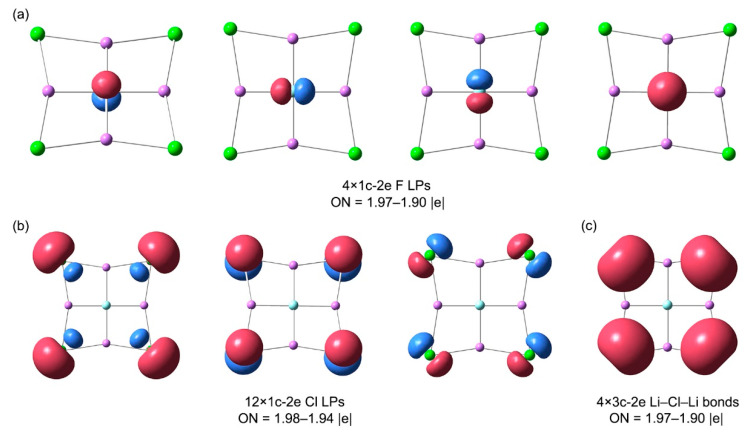
Chemical bonding pattern for FLi_4_Cl_4_^−^ (**1**) cluster, according to the adaptive natural density partitioning (AdNDP) analysis. Occupation numbers (ONs) are shown: (**a**) 4 LPs of F, (**b**) 12 LPs of 4 Cl atoms, and (**c**) 4 Li–Cl–Li 3c-2e bonds.

**Figure 8:**
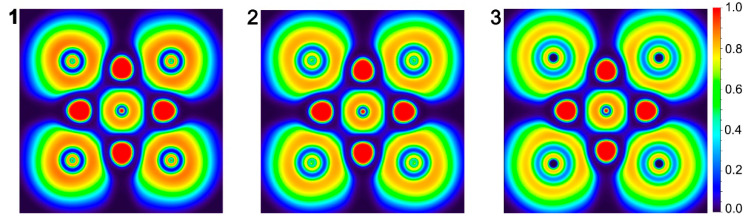
Electron localization function (ELF) plots of the ptF **1**, **2**, and **3** clusters.

The bonding pattern of cluster **1** was also investigated using the adaptive natural density partition (AdNDP) method, which is widely used in discussions of localized multicenter chemical bonding [44]. AdNDP analysis not only recovers classical Lewis bonding elements (lone pairs (LPs) and 2c-2e bonds) but also delocalized *n*c-2e (*n* ≥ 3) bonds. The AdNDP bonding scheme of **1** is depicted in Figure 7, which provides a relatively simple and intuitive bonding picture. There are four 1c-2e LPs for the central F atom, 12 LPs for four periphery Cl atoms, and four 3c-2e Li–Cl–Li σ bonds. All occupation numbers (ONs) range from 1.98 to 1.90|e|, which is close to the ideal 2.00|e|. It should be noted that the contributions of the Cl atoms in these bonds are extremely important. If these Li–Cl–Li σ bonds are approximated as the LPs of the Cl atoms, the ON value can reach 1.85|e|. In other words, these 3c-2e bonds are basically dominated by the LPs of Cl atoms. Herein, the AdNDP bonding pattern is in complete agreement with the above conclusion obtained by CMO analysis. As shown in Figure 8, the bonding patterns of **1**–**3** are also perfectly borne out by the calculated electron localization functions (ELFs) [45]. The LP electrons feature of ptF centers in the figure is obvious. The strong alkali metal properties of Li and the strong electronegativity of halogen atoms make the system completely dominated by ionic bonds.

The multicenter ionic bonding is the key factor for the stability of the ptF **1**–**3** clusters. To further quantitatively reveal the ionic bonding characters of the ptF **1**–**3** clusters, we performed the interacting quantum atoms (IQA) analysis [46,47,48]. IQA can offer an accurate description of the FLi_4_X_4_^−^ (X = Cl, Br, I) clusters. The interatomic interaction energy ($V_{IQA}^{\mathrm{int}}$) includes electrostatic interaction energy ($V_{C}^{\mathrm{int}}$) and covalent interaction energy ($V_{XC}^{\mathrm{int}}$). As listed in Table 1, the absolute values of $V_{C}^{\mathrm{int}}$ (F–Li) and $V_{C}^{\mathrm{int}}$ (Li–Cl) of cluster **1** are 145.28 and 119.91 kcal mol^−1^, respectively, which are much greater than the corresponding $V_{XC}^{\mathrm{int}}$ (F–Li) and $V_{XC}^{\mathrm{int}}$ (Li–Cl) values 10.59 and 13.08 kcal mol^−1^. Thus, both F–Li and Li–Cl interactions are completely dominated by ionic bonding. Note that the $V_{IQA}^{\mathrm{int}}$ (Li–Li) is + 98.65 kcal mol^−1^, suggesting the interaction between two adjacent Li atoms is electrostatic repulsion. The bonding characters of **2** and **3** are basically similar to those of **1**. The IUPAC definition of coordination number refers to “the number of other atoms that are directly bonded to that particular atom”. Thus, FLi_4_X_4_^−^ (X = Cl, Br, I) are truly ptF species stabilized by multicenter ionic bonding.

**Figure 9:**
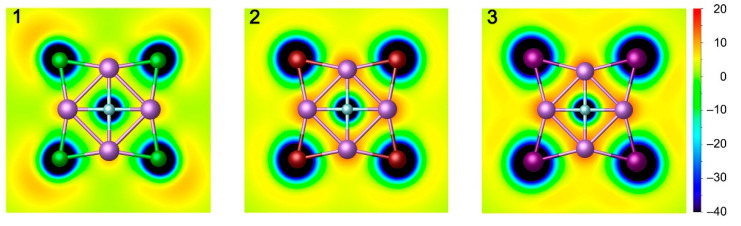
Color-filled maps of NICS(0)_zz_ (in ppm) for **1**–**3**. Negative values indicate aromaticity.

In general, perfectly flat molecules are always associated with aromaticity. Geometrically, high symmetry (*D*_4*h*_) equalizes the corresponding bond distances in the **1**–**3** systems. Are these ptF anions σ-aromatic? To probe the aromaticity, we performed the nucleus-independent chemical shifts (NICS) analysis [49]. NICS in the z-direction, NICS_zz_, is one suitable criterion for aromaticity. To intuitively observe the σ aromaticity, the color-filled maps of NICS(0)_zz_ of **1**–**3** are plotted in Figure 9. In fact, the regions with negative NICS(0)_zz_ values are only within the very small regions around the F and X atoms in the graph. The alkali Li metals, which have almost no valence electrons, cannot participate in electron delocalization. High ionization greatly weakens the contribution of aromaticity to the stability of the system. In other words, the stabilities of ptF centers in **1**–**3** are dominated by multicenter ionic bonding, rather than the σ aromaticity.

### 2.3. Superhalogen Anion Characters

These planar 40 ve closed-shell FLi_4_X_4_*^−^* (**1**–**3**) clusters are possible to have extremely high VDEs, that is, to serve as superhalogen anions, due to their stable closed-shell electronic configurations and the anticipated sizable HOMO–LUMO gaps. We want to know that if they have superhalogen anion characters, the easiest way to find this is to calculate their first vertical detachment energies (VDEs). The VDEs were calculated as the energy differences between the anion and the neutral at the ground-state structures of the anions. At the singlet CCSD(T)/def2-TZVPP//PBE0-D3(BJ)/def2-TZVPP level, the calculated first VDEs were 7.37, 6.94, and 6.30 eV for **1**–**3**, respectively. Vertical one-electron detachment energies of **1**–**3** were also calculated at the outer valence Green’s function (OVGF) level using the anion structures at PBE0-D3(BJ)/def2-TZVPP [50]. At the OVGF/def2-TZVPP level, the first VDEs were 7.44, 7.09, and 6.33 eV, which are very close to those of CCSD(T) data. Note that the values of the first VDEs of **1**–**3** are positively correlated with the electronegativity of the auxiliary X atoms. These VDE values are clearly greater than the EA of Cl (3.61 eV). According to the definition, the ptF FLi_4_X_4_*^−^* (X = Cl, Br, I) species belong to the superhalogen anions. Thus, using Li as the ligands and high electronegative Cl, Br, and I as outer auxiliary atoms is an effective strategy in the designs of novel ptF superhalogen anions.

## 3. Methods

The GM searches for FLi_4_X_4_^−^ (X = Cl, Br, I) clusters were conducted at the density functional theory (DFT) level using the Coalescence Kick (CK 1.0) search program, aided with manual structural constructions [51]. The CK method is based on randomly placing the atoms constituting the cluster in a large Cartesian box, far enough apart, and then pushing them toward the center of mass until they coalesce up to one fragment. Then, normal geometry optimization is applied to it. If not, the CK procedure is applied to the fragmented structure; that is, all the fragments are pushed to the center of mass simultaneously. The box size is 4× (sum of covalent radii of atoms) in all three linear dimensions. The magnitude of the shift is 0.2 Å in the current version of the CK program. More detailed descriptions can be found in the literature [52,53]. The initial screening of the singlet and triplet states was carried out at the PBE0/def2-SVP level. The candidate low-lying isomers were reoptimized at the PBE0-D3(BJ)/def2-TZVPP level. The addition of D3(BJ) correction energy can improve the description of the non-covalent interactions of the FLi_4_X_4_^−^ (X = Cl, Br, I) clusters and better evaluate the structures and relative energies. The harmonic vibrational frequencies were analyzed at the same level to confirm their minimal nature on the respective potential energy surfaces. The energies of the top five low-lying isomers were further improved by the CCSD(T)/def2-TZVPP single-point calculations based on the PBE0-D3(BJ)/def2-TZVPP optimized geometries. To evaluate the dynamic stabilities of ptF FLi_4_X_4_^−^ (X = Cl, Br, I), BOMD simulations (at 298K for 100 ps) were implemented employing the CP2K software with the GTH-PBE pseudopotentials and the DZVP-MOLOPTSR-GTH basis set (https://www.cp2k.org/). NBO, CMOs, AdNDP, and ELF analyses were performed to gain insights into the bonding of these ptF species. To probe the aromatic characters of these ptF species, the NICS were calculated at the molecular plane. The VDEs were comparatively calculated with the OVGF methods. Orbital compositions were analyzed using Multiwfn [54] (http://sobereva.com/multiwfn/, accessed on 28 October 2024). All electronic structure calculations were performed with Gaussian 16 [55]. The interacting quantum atoms (IQAs) analysis was performed using the ADF (2023) program [56].

## 4. Conclusions

Using Li as the ligands and Cl, Br, and I as outer auxiliary bridges, we have computationally designed the first series of unique star-like ptF FLi_4_X_4_^−^ (X = Cl, Br, I) superhalogen anions. The bonding in the systems features the peripheral four Li–X–Li 3c-2e σ bonds, as well as the eight-electron shell associated with the ptF center. This bonding pattern manages to stabilize the ptF FLi_4_X_4_^−^ anions. These viable ptF species are stabilized by ionic bonding, not σ aromaticity. Very high first VDEs are predicted for the species, 7.37, 6.94, and 6.30 eV for X = F, Cl, and Br, consistent with their nature as superhalogen anions. Large HOMO–LUMO energy gaps are also calculated, 7.49, 7.05, and 6.46 eV for X = F, Cl, and Br. The current work built an important link between the ptF chemistry and superhalogen anions. Experimental characterizations of these predicted ptF superhalogen anions are invited to effectively enrich the chemistries of planar hypercoordinate fluorines and superhalogens.

## Figures and Tables

**Table 1:** Energy components of IQA for the *D*_4*h*_ FLi_4_X_4_^−^ (X = Cl, Br, I) systems at the PBE0/TZ2P level; $V_{IQA}^{\mathrm{int}}$, $V_{C}^{\mathrm{int}}$, and $V_{XC}^{\mathrm{int}}$ are the interatomic IQA interaction energy and their coulombic and exchange-correlation energy components, respectively, in kcal mol^−1^.

	FLi_4_Cl_4_^−^	FLi_4_Br_4_^−^	FLi_4_I_4_^−^
$V_{IQA}^{\mathrm{int}}$ (F–Li)	−155.87	−155.23	−154.59
$V_{C}^{\mathrm{int}}$ (F–Li)	−145.28 (93.21%)	−144.69 (93.21%)	−144.23 (93.30%)
$V_{XC}^{\mathrm{int}}$ (F–Li)	−10.59 (6.79%)	−10.54 (6.79%)	−10.36 (6.70%)
$V_{IQA}^{\mathrm{int}}$ (Li–Li)	+98.65	+98.49	+97.45
$V_{C}^{\mathrm{int}}$ (Li–Li)	+98.70 (99.95%)	+98.54 (99.95%)	+97.51 (99.94%)
$V_{XC}^{\mathrm{int}}$ (Li–Li)	−0.05 (0.05%)	−0.05 (0.05%)	−0.06 (0.06%)
$V_{IQA}^{\mathrm{int}}$ (Li–X)	−132.99	−128.69	−122.31
$V_{C}^{\mathrm{int}}$ (Li–X)	−119.91 (90.16%)	−116.82 (90.78%)	−111.12 (90.85%)
$V_{XC}^{\mathrm{int}}$ (Li–X)	−13.08 (9.84%)	−11.87 (9.22%)	−11.19 (9.15%)

## Data Availability

Data are contained within this article and Supplementary Materials.

## Supplementary Materials

The following supporting information can be downloaded at https://www.mdpi.com/article/10.3390/molecules29235810/s1, Table S1: The lowest vibrational frequency at eight classical theoretical levels for the global-minimum structure **1**–**3**; Table S2: The relative energies of CCSD(T)/aug-cc-pVQZ for FLi_4_Cl_4_^−^ structures with the central F atom located 0.0, 0.1, 0.2, 0.3 and 0.4 Å above the plane; Table S3: Composition analysis of canonical molecular orbitals (CMOs) for **1** at the PBE0/def2-TZVPP level; Figure S1: Optimized global-minimum structures **1**, **2**, **3** and their four low-lying isomers (***n*B**−***n*E**) at the PBE0-D3(BJ)/aug-cc-pVQZ (aug-cc-pVQZ(PP) for I) level. Relative energies are listed in kcal mol^−1^ at the PBE0-D3(BJ)/aug-cc-pVQZ level. The CCSD(T)/aug-cc-pVQZ//PBE0-D3(BJ)/aug-cc-pVQZ relative energies for **1** and **1B** are also listed in square brackets; Figure S2: Calculated F1−Li2−Li3−Li4 dihedral angles of **1**−**3** clusters during the BOMD simulations at 298K; Cartesian coordinates of top five low-lying isomers of FLi_4_X_4_^−^ (X = Cl, Br, I) at PBE0-D3(BJ)/def2-TZVPP.
