# Supplementary material for: FLi4X4− (X = Cl, Br, I): Superhalogen Anions with Planar Tetracoordinate Fluorine
Source: Molecules. 2024 Dec 9;29(23):5810. doi: 10.3390/molecules29235810 (PMC11643589; doi:10.3390/molecules29235810)
Supplement: Supplementary file 1 [file molecules-29-05810-s001.zip › molecules-3312798-supplementary.pdf]

SUPPORTING INFORMATION

$FLi_4X_4^-$ (X = Cl, Br, I): Superhalogen Anions with Planar Tetracoordinate Fluorine

Yong-Xia Li [1,*], Li-Xia Bai [2] and Jin-Chang Guo [2,*]

[1] Department of Chemistry, Xinzhou Normal University, Xinzhou 034000, China
[2] Institute of Molecular Science, Shanxi University, Taiyuan 030006, China
* Correspondence: liyongxia0126@163.com (Y.-X.L.); guojc@sxu.edu.cn (J.-C.G.)

Supporting Information

Table S1. The lowest vibrational frequency at eight theoretical levels for the global-minimum structures of $FLi_4X_4^-$ (X = Cl, Br, I) (**1–3**).

Table S2. The relative energies of CCSD(T)/aug-cc-pVQZ for $FLi_4Cl_4^-$ structures with the central F atom located 0.0, 0.1, 0.2, 0.3 and 0.4 Å above the plane.

Table S3. Composition analysis of canonical molecular orbitals (CMOs) for the GM (**1**) structure of $FLi_4Cl_4^-$ at the PBE0/def2-TZVPP level.

Figure S1. Optimized global-minimum structures **1**, **2**, **3** and their four low-lying isomers (***n*B–*n*E**) at the PBE0-D3(BJ)/aug-cc-pVQZ (aug-cc-pVQZ(PP) for I) level. Relative energies are listed in kcal mol^{-1} at the PBE0-D3(BJ)/aug-cc-pVQZ level. The CCSD(T)/aug-cc-pVQZ//PBE0-D3(BJ)/aug-cc-pVQZ relative energies for **1** and **1B** are also listed in square brackets.

Figure S2. Calculated F1−Li2−Li3−Li4 dihedral angles of **1–3** clusters during the BOMD simulations at 298K.

Cartesian coordinates of top five low-lying isomers of $FLi_4X_4^-$ (X = Cl, Br, I) at PBE0-D3(BJ)/def2-TZVPP.

Table S1. The lowest vibrational frequency at eight theoretical levels for the global-minimum structures of $FLi_4X_4^-$ (X = Cl, Br, I) (**1-3**).

Theoretical level	Lowest vibrational frequency (cm^{-1})		
	$FLi_4Cl_4^-$	$FLi_4Br_4^-$	$FLi_4I_4^-$
PBE0-D3(BJ)/def2-TZVPP	33.2	18.2	10.9
B3PW91-D3(BJ)/def2-TZVPP	33.1	17.8	10.8
B3LYP-D3(BJ)/def2-TZVPP	32.2	16.7	3.2
B2PLYP-D3(BJ)/def2-TZVPP	33.5	17.8	7.4
MP2/def2-TZVPP	35.2	19.0	12.1
CCSD/def2-TZVPP	34.6	18.9	11.5
ωB97X-D/def2-TZVPP	30.2	16.9	6.0
TPSSh/def2-TZVPP	30.2	19.3	3.9

Table S2. The relative energies of CCSD(T)/aug-cc-pVQZ for $FLi_4Cl_4^-$ structures with the central F atom located 0.0, 0.1, 0.2, 0.3 and 0.4 Å above the plane.

Above the plane (Å)	Relative energy (kcal mol^{-1})
0.0	0.0
0.1	0.2
0.2	0.6
0.3	1.4
0.4	2.7

Table S3. Composition analysis of canonical molecular orbitals (CMOs) for the GM (**1**) structure of $FLi_4Cl_4^-$ at the PBE0/def2-TZVPP level.

CMO	F (%)		Li_4 (%)		Cl_4 (%)	
	s/p	total	s/p	total	s/p	total
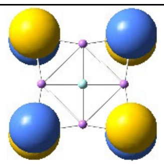 HOMO (b_{1u})	0.00/0.00	0.00	0.00/0.00	0.00	0.0/99.92	99.92
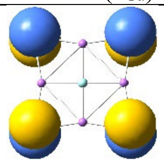 HOMO-1 (e_g)	0.00/0.00	0.00	0.00/4.32	4.32	0.00/97.76	97.76
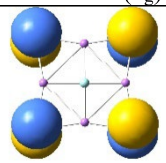 HOMO-1′ (e_g)	0.00/0.00	0.00	0.00/4.32	4.32	0.00/97.76	97.76
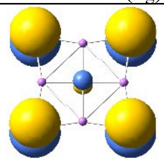 HOMO-2 (a_{2u})	0.00/1.16	1.16	0.00/3.68	3.68	0.00/95.08	95.08
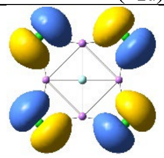 HOMO-3 (a_{2g})	0.00/0.00	0.00	0.00/0.44	0.44	0.00/98.96	98.96
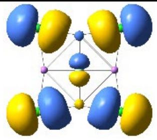 HOMO-4 (e_u)	0.00/2.95	2.95	2.58/0.22	2.80	0.00/93.56	93.56

CMO	F (%)		Li_4 (%)		Cl_4 (%)	
	s/p	total	s/p	total	s/p	total
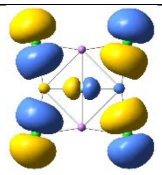 HOMO-4′ (e_u)	0.00/2.95	2.95	2.58/0.22	2.80	0.00/**93.56**	**93.56**
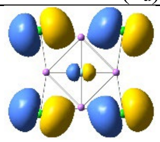 HOMO-5 (e_u)	0.00/1.48	1.48	0.00/0.24	0.24	0.00/**91.28**	**91.28**
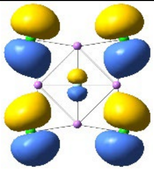 HOMO-5′ (e_u)	0.00/1.48	1.48	0.00/0.24	0.24	0.00/**91.28**	**91.28**
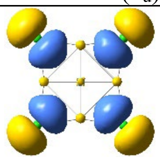 HOMO-6 (a_{1g})	0.38/0.00	0.00	3.60/0.84	4.44	0.00/95.00	95.00
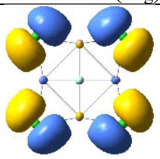 HOMO-7 (b_{1g})	0.00/0.00	0.00	3.16/2.56	5.72	0.00/94.00	94.00
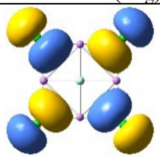 HOMO-8 (b_{2g})	0.00/0.00	0.00	0.00/1.16	1.16	0.44/97.80	98.24
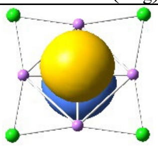 HOMO-9 (a_{2u})	0.00/97.24	97.24	0.00/2.08	2.08	0.00/0.64	0.64
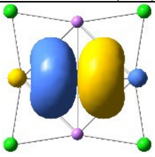 HOMO-10 (e_u)	0.00/93.97	93.97	2.60/0.69	3.29	0.32/0.90	1.22

CMO	F (%)		Li_4 (%)		Cl_4 (%)	
	s/p	total	s/p	total	s/p	total
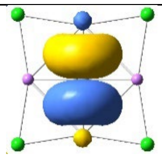 HOMO-10′(e_u)	0.00/93.97	93.97	2.60/0.69	3.29	0.32/0.90	1.22
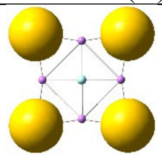 HOMO-11 (a_{1g})	0.06/0.00	0.06	3.08/0.12	3.20	96.72/0.00	96.72
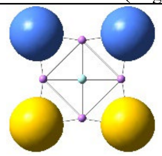 HOMO-12 (e_u)	0.00/0.06	0.06	1.70/3.86	5.76	94.04/0.06	94.10
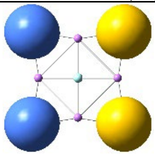 HOMO-12′ (e_u)	0.00/0.06	0.06	1.70/3.86	5.76	94.04/0.06	94.10
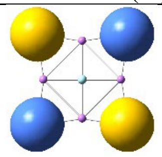 HOMO-13 (b_{2g})	0.00/0.00	0.00	0.00/6.60	6.60	93.20/0.08	93.28
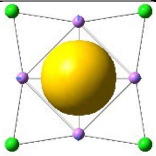 HOMO-14 (a_{1g})	94.43/0.00	94.43	0.00/5.52	5.52	0.00/0.00	0.00

Figure S1. Optimized global-minimum structures **1**, **2, 3** and their four low-lying isomers (***n*****B–*n*****E**) at the PBE0-D3(BJ)/aug-cc-pVQZ (aug-cc-pVQZ(PP) for I) level. Relative energies are listed in kcal mol^{-1} at the PBE0-D3(BJ)/aug-cc-pVQZ level. The CCSD(T)/aug-cc-pVQZ//PBE0-D3(BJ)/aug-cc-pVQZ relative energies for **1** and **1B** are also listed in square brackets.

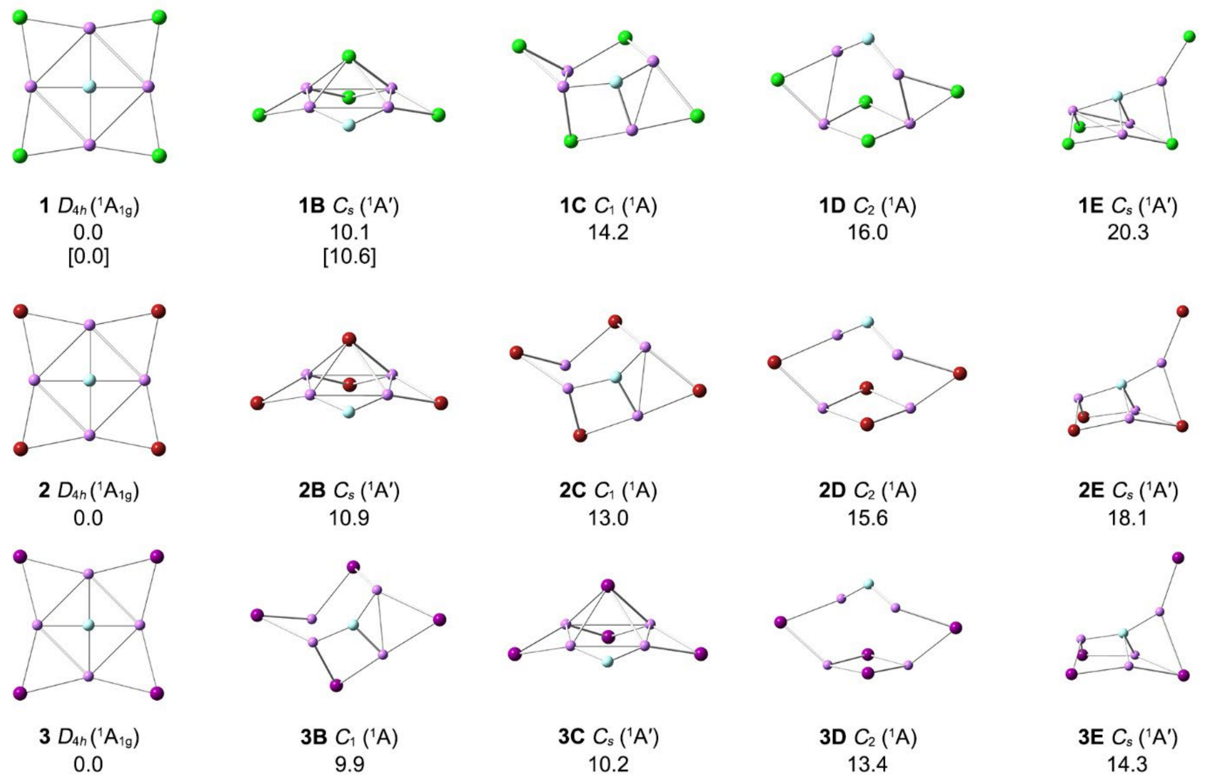

Figure S2. Calculated F1−Li2−Li3−Li4 dihedral angles of **1–3** clusters during the BOMD simulations at 298K.

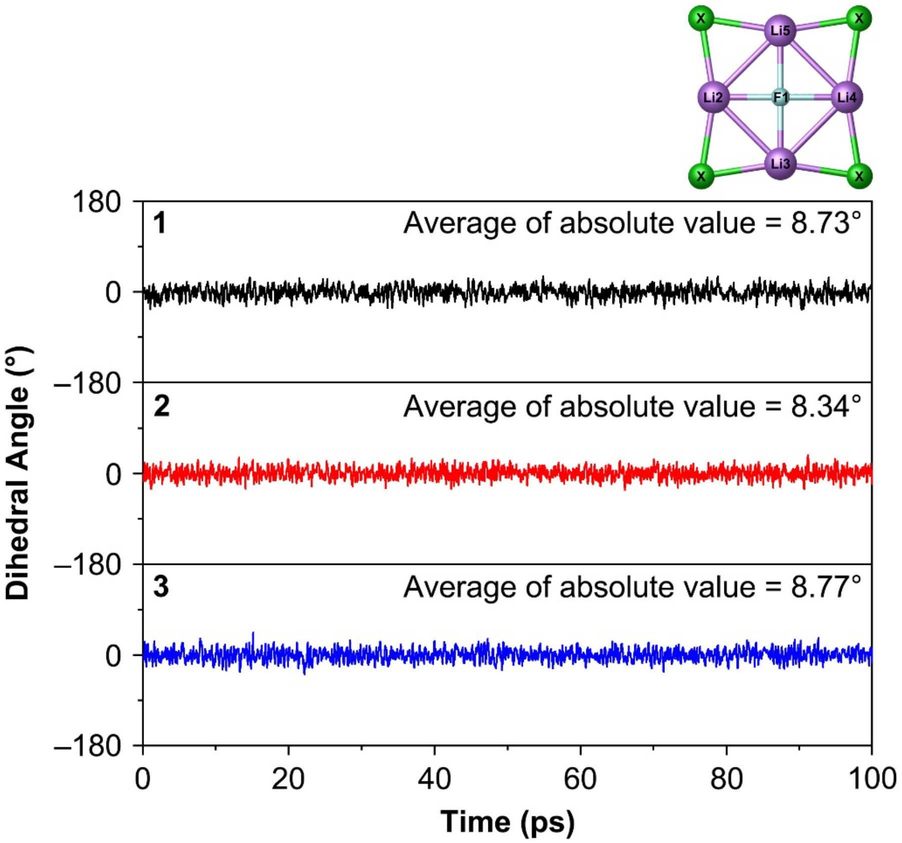

Cartesian coordinates of top five low-lying isomers of $FLi_4X_4^-$ (X = Cl, Br, I) at PBE0-D3(BJ)/def2-TZVPP.

1 $FLi_4Cl_4^-$ (D_{4h}, $^1A_{1g}$)

Cl	2.21887300	-2.21887300	0.00000000
Cl	-2.21887300	-2.21887300	0.00000000
Cl	-2.21887300	2.21887300	0.00000000
Cl	2.21887300	2.21887300	0.00000000
F	0.00000000	0.00000000	0.00000000
Li	0.00000000	1.87075600	0.00000000
Li	1.87075600	0.00000000	0.00000000
Li	0.00000000	-1.87075600	0.00000000
Li	-1.87075600	0.00000000	0.00000000

1B (C_s, $^1A'$)

F	-0.78687500	-2.70588600	0.00000000
Li	-0.76437000	-1.59282900	1.31614900
Li	-0.76437000	-1.59282900	-1.31614900
Li	0.28591900	1.06621600	1.42336700
Li	0.28591900	1.06621600	-1.42336700
Cl	0.28591900	-0.52661400	-3.00438800
Cl	-1.48738700	0.36379700	0.00000000
Cl	0.28591900	-0.52661400	3.00438800
Cl	1.50099500	2.30782300	0.00000000

1C (C_1, ^1A)

F	0.41239400	-0.48954900	1.49230100
Li	1.33728500	-1.34801300	0.16081700
Li	-1.35973100	0.53454200	-0.92713100
Li	1.30271500	0.97019900	0.80188300
Li	-1.21145500	-1.01206500	1.04665000
Cl	3.22451300	-0.17390100	0.21692300
Cl	0.10107300	2.20206300	-0.66312500
Cl	-3.03780300	0.15340800	0.57805300
Cl	-0.51825200	-1.77145400	-1.11287300

1D (C_2, ^1A)

F	0.00000000	0.00000000	2.13176400
Li	1.08137800	-1.01758400	-1.05198700

Li	-1.08137800	1.01758400	-1.05198700
Li	0.00000000	1.45662200	1.23000700
Li	0.00000000	-1.45662200	1.23000700
Cl	-1.82120600	2.55170100	0.37351600
Cl	-1.24047600	-1.24765200	-0.96922200
Cl	1.24047600	1.24765200	-0.96922200
Cl	1.82120600	-2.55170100	0.37351600

1E (C_s, $^1A'$)

F	-0.62197200	-0.24114400	0.00000000
Li	0.71027900	-0.07385100	1.32626500
Li	-0.27228800	-2.06993200	0.00000000
Li	0.71027900	-0.07385100	-1.32626500
Li	-1.00724200	1.58728700	0.00000000
Cl	0.71027900	-2.16612400	2.04778000
Cl	0.71027900	-2.16612400	-2.04778000
Cl	1.52696900	1.64135200	0.00000000
Cl	-2.64313600	2.92979900	0.00000000

2 $FLi_4Br_4^-$ (D_{4h}, $^1A_{1g}$)

F	0.00000000	0.00000000	0.00000000
Li	0.00000000	1.88514300	0.00000000
Li	1.88514300	0.00000000	0.00000000
Li	0.00000000	-1.88514300	0.00000000
Li	-1.88514300	0.00000000	0.00000000
Br	-2.35323900	2.35323900	0.00000000
Br	-2.35323900	-2.35323900	0.00000000
Br	2.35323900	-2.35323900	0.00000000
Br	2.35323900	2.35323900	0.00000000

2B (C_s, $^1A'$)

F	-0.92174600	-2.92096300	0.00000000
Li	-0.89895800	-1.85866500	1.34608000
Li	-0.89895800	-1.85866500	-1.34608000
Li	0.23429800	0.90800400	1.48756000
Li	0.23429800	0.90800400	-1.48756000
Br	0.23429800	-0.78847500	-3.19921800
Br	-1.72910600	0.28100500	0.00000000
Br	0.23429800	-0.78847500	3.19921800
Br	1.61147200	2.21001900	0.00000000

2C (C_1, ^{1}A)

F	0.42791200	-0.43988700	1.59633100
Li	1.39149800	-1.41247700	0.38871100
Li	-1.40376400	0.43731600	-0.91585100
Li	1.33696100	0.99460400	0.92536300
Li	-1.20580600	-0.99407800	1.25938100
Br	3.46290600	-0.21953900	0.48137700
Br	0.15974800	2.24892800	-0.78982300
Br	-3.19838200	0.20504700	0.74675700
Br	-0.54449800	-2.03778300	-0.99087700

2D (C_2, ^{1}A)

F	0.00000000	0.00000000	2.30628700
Li	1.10154900	-1.10344100	-0.97072600
Li	-1.10154900	1.10344100	-0.97072600
Li	0.00000000	1.47096100	1.44551800
Li	0.00000000	-1.47096100	1.44551800
Br	-1.90721700	2.75126100	0.56145100
Br	-1.38119500	-1.31545400	-0.89867000
Br	1.38119500	1.31545400	-0.89867000
Br	1.90721700	-2.75126100	0.56145100

2E (C_s, ^{1}A′)

F	-0.50894200	-0.24794000	0.00000000
Li	0.77113700	-0.03203400	1.36027700
Li	-0.25487400	-2.08174600	0.00000000
Li	0.77113700	-0.03203400	-1.36027700
Li	-1.09604600	1.51858400	0.00000000
Br	0.77113700	-2.25226400	2.21105000
Br	0.77113700	-2.25226400	-2.21105000
Br	1.58814300	1.87738800	0.00000000
Br	-3.01594700	2.74465800	0.00000000

3 $FLi_4I_4^-$ (D_{4h}, $^1A_{1g}$)

F	0.00000000	0.00000000	0.00000000
Li	0.00000000	1.89901700	0.00000000
Li	1.89901700	0.00000000	0.00000000
Li	0.00000000	-1.89901700	0.00000000
Li	-1.89901700	0.00000000	0.00000000
I	-2.53494800	2.53494800	0.00000000

I	-2.53494800	-2.53494800	0.00000000
I	2.53494800	-2.53494800	0.00000000
I	2.53494800	2.53494800	0.00000000

3B (C_s, $^1A'$)

F	-1.02179300	-3.03072300	0.00000000
Li	-1.00657800	-2.03286800	1.38172100
Li	0.21130300	0.87075400	-1.57465400
Li	-1.00657800	-2.03286800	-1.38172100
Li	0.21130300	0.87075400	1.57465400
I	0.21130300	-0.95870800	3.47567600
I	0.21130300	-0.95870800	-3.47567600
I	-1.99766600	0.34912300	0.00000000
I	1.83860500	2.21450300	0.00000000

3C (C_1, ^1A)

F	-1.02179300	-3.03072300	0.00000000
Li	-1.00657800	-2.03286800	1.38172100
Li	0.21130300	0.87075400	-1.57465400
Li	-1.00657800	-2.03286800	-1.38172100
Li	0.21130300	0.87075400	1.57465400
I	0.21130300	-0.95870800	3.47567600
I	0.21130300	-0.95870800	-3.47567600
I	-1.99766600	0.34912300	0.00000000
I	1.83860500	2.21450300	0.00000000

3D (C_s, $^1A'$)

F	-0.33301900	-0.20271200	0.00000000
Li	0.88661900	0.06734800	1.39959700
Li	-0.20297200	-2.04126000	0.00000000
Li	0.88661900	0.06734800	-1.39959700
Li	-1.15005300	1.45730400	0.00000000
I	1.69778900	2.23888000	0.00000000
I	-3.43826100	2.46089400	0.00000000
I	0.88661900	-2.31996100	2.42989800
I	0.88661900	-2.31996100	-2.42989800

3E (C_2, ^1A)

F	0.00000000	0.00000000	2.40566200
Li	1.12798000	-1.21310200	-0.98347800

Li	-1.12798000	1.21310200	-0.98347800
Li	0.00000000	1.49097800	1.59908500
Li	0.00000000	-1.49097800	1.59908500
I	-2.03077900	3.01246400	0.69074900
I	-1.57683200	-1.40764300	-0.92984900
I	1.57683200	1.40764300	-0.92984900
I	2.03077900	-3.01246400	0.69074900
